# Development of a rapid LFA test based on direct RT-LAMP for diagnosis of SARS-CoV-2

**DOI:** 10.1016/j.plabm.2024.e00437

**Published:** 2024-10-18

**Authors:** Negar Sadeghi, Neda Shirazi, Moein Dehbashi, Bahareh Maleki, William C. Cho, Zohreh Hojati

**Affiliations:** a Division of Genetics, Department of Cell and Molecular Biology and Microbiology, Faculty of Biological Science and Technology, University of Isfahan, Isfahan, Postal Code: 81746-73441, Iran; b Department of Clinical Oncology, Queen Elizabeth Hospital, Hong Kong SAR, China

**Keywords:** SARS-CoV-2, COVID-19, RT-qPCR, RT-LAMP, LFA

## Abstract

**Introduction:**

In response to the rapid spread of the SARS-CoV-2 virus, we developed a rapid molecular approach to diagnose COVID-19 without the need for RNA extraction.

**Methods:**

The study utilized two molecular methods, RT-qPCR and colorimetric RT-LAMP, to diagnose the RdRp and ORF8 genes, respectively, in oro-nasopharyngeal swabs. Due to the high sequence diversity of ORF8 in SARS-CoV and SARS-CoV-2, it has been identified as a suitable target for virus detection. The RT-LAMP method was also carried out directly on heat-treated swab samples. The strip tests were made using gold nanoparticles and combined with the RT-LAMP for further analysis.

**Results:**

The results showed that the isothermal amplification method had a sensitivity of 95 % (95 % C.I.: 86.08 %–98.96 %) and a specificity of 75 % (95 % C.I.: 19.41 %–99.37 %). The RT-LAMP-LFA method was able to distinguish positive and negative samples with 100 % sensitivity (95 % C.I.: 91.96–100) and 77.27 % specificity (95 % C.I.: 54.63–92.18). This method only required heating swab samples for 10 min at 65 °C before the RT-LAMP reaction.

**Conclusion:**

By utilizing the RT-LAMP in combination with the LFA, it is possible to diagnose SARS-CoV-2 rapidly without the need for RNA extraction. The entire process from sample collection to test interpretation takes only 75–90 min, and the results can be interpreted by untrained individuals with the naked eye. By employing the ORF8 gene as a diagnostic target and eliminating the need for RNA extraction, the direct RT-LAMP-LFA method achieves a significant breakthrough that was not previously reported.

## Highlights

• A newly developed rapid COVID-19 diagnostic test that doesn't require RNA extraction.
• Comparison of RT-qPCR and colorimetric RT-LAMP to detect SARS-CoV-2 in swab samples.
• ORF8 is a suitable target for virus detection due to its high sequence diversity.
• Direct RT-LAMP-LFA achieves a rapid and user-friendly diagnostic process.
• Sampling to test results takes 75–90 min, interpretable by the untraineds.

## Graphical abstract

**Figure:**
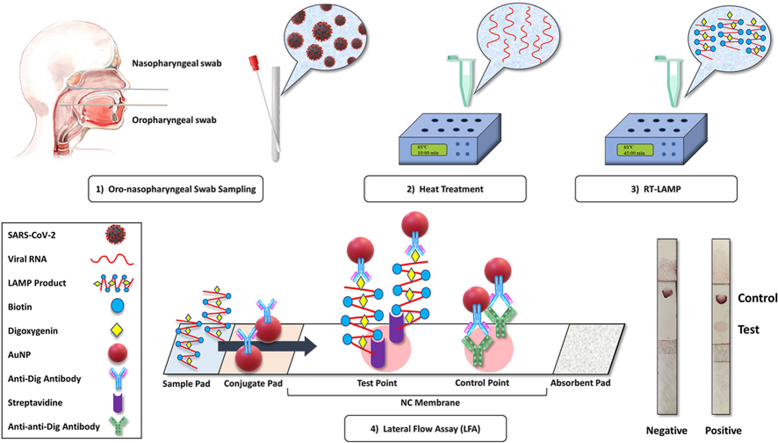
A schematic representation of the direct RT-LAMP-LFA performance for the diagnosis of SARS-CoV-2.

## 1 Introduction

COVID-19, caused by SARS-CoV-2, is a rapidly expanding viral disease, based on WHO reports by December 19, 2023, has resulted in more than 770 million proven cases including around 6.9 million deaths [1]. According to this severe threat, early, rapid, and accurate diagnosis of SARS-CoV-2 is critical to prevent further spread and control of the pandemic [2]. Currently, the quantitative reverse transcription-polymerase chain reaction (RT-qPCR) has been considered a gold standard method for the detection of COVID-19 [3]. However, the need for trained technicians and expensive instruments restricts the use of this method in less-developed areas of the world [4]. Another weakness of the present assay is the probability of false-positive results that can be due to existing contamination [5]. Loop-mediated isothermal amplification (LAMP) is a type of isothermal nucleic acid amplification technique (iNAAT) that can prevail over these limitations. In comparison to PCR, LAMP is more effective due to its capacity to amplify the intended DNA without interference from non-target sequences. This is achieved without the need for multiple temperature cycles, extended reaction times, or intricate laboratory setups, all of which can contribute to amplification errors and longer processing durations. Lateral flow assay (LFA) is a paper-based diagnostic tool that is highly regarded for its speed and simplicity in performing tests [6]. In this assay, the test result appears expeditiously as positive/negative signals, easily observable to the naked eye and untrained people [7]. Therefore, the collaboration of NAATs with LFA can assist in detecting the target nucleic acid quickly and efficiently [8]. In the current study, our primary aim was to compare the detection accuracy of colorimetric RT-LAMP with RT-qPCR. Hence, we first used the colorimetric RT-LAMP in two different manners, targeting the ORF8 gene: (i) Based on viral isolated RNA from swabs and (ii) directly without the need for RNA extraction. Then to achieve a rapid diagnostic method, we designed a gold nanoparticle-based LFA to rapidly detect SARS-CoV-2 through the visual detection of RT-LAMP products. Finally, the isothermal amplification method in combination with LFA was evaluated on a lateral flow strip.

## 2 Materials and methods

### 2.1 Clinical specimens

All combined oro-nasopharyngeal swab specimens were remaining specimens from viral diagnostic testing that were either kept in physiological serum or viral transport media (VTM). The study population consisted of 130 samples, 64 of which were gathered from Aria Medical Laboratory and 66 from Melal Medical Laboratory, Isfahan, Iran. The samples were stored at −70 °C for further processing.

### 2.2 RNA extraction for comparison of RT-qPCR method with RT-LAMP

Viral RNA was isolated from the swab samples using the MaxCell kit (BioMaxCell Co., Tehran, Iran) based on the manufacturer's protocol. The RT-qPCR assay was performed in one step using PowerChek™ 2019-nCoV Real-time PCR Kit (Kogene Biotech, Korea), according to the manufacturer's instructions. This method was operated on Applied Biosystems™ StepOne™ Real-Time PCR System (Thermo Fisher Scientific I, USA). The real-time PCR program was applied with a 30-min reverse transcription step at 50 °C and a 10 min initial denaturation step at 95 °C subsequently by 40 amplification cycles with a denaturation temperature of 95 °C for 10 s, annealing and extension temperatures of 60 °C for 1 min.

### 2.3 Colorimetric RT-LAMP assay

Based on the recommended protocol, WarmStart Colorimetric LAMP 2X Master Mix (NEB, MA, USA) was used. The reaction solution contains a visible pH indicator that detects amplification based on the production of protons and the subsequent drop in pH that occurs from the extensive DNA polymerase activity in a LAMP reaction, producing a change in solution color from pink to yellow. A published LAMP primer set targeting the ORF8 gene of SARS-CoV-2 was selected [9] which is shown in Table S1. The RT-LAMP reactions used in this study were as follows: 6 μl Ready-mix, 1.6 mM FIP primer, 1.6 mM BIP primer, 0.2 mM F3 primer, 0.2 mM B3 primer, 0.4 mM LF primer, 0.4 mM LB primer and 1 μl extracted RNA to a total volume reaction of 12.5 μl. In several tests, 1 μl of the crude samples (swabs in physiological serum; pre-incubated at 65 °C for 10 min) was substituted for the extracted RNA. The reactions were performed at a consistent temperature of 65 °C for 60 min. The nuclease-free water and a positive clinical sample confirmed by both RT-LAMP and RT-qPCR were considered negative and positive controls, respectively. 2 % agarose gel electrophoresis was performed to visualize LAMP products and verify true positives.

### 2.4 Limit of detection

The limit of detection (LoD) of colorimetric RT-LAMP and RT-qPCR methods was evaluated using an isolated RNA from a positive sample verified by RT-qPCR and colorimetric RT-LAMP. The RNA was serially diluted ranging from 40 to 4 $\times 10^{‐6}$ ng/μl. Finally, the results were compared between colorimetric RT-LAMP and RT-qPCR.

### 2.5 Gold nanoparticle (AuNP) synthesis

For AuNP synthesis, 100 mM HAuCl_4_.3H_2_O and 38.8 mM trisodium citrate dihydrate were used. After the chemical synthesis, the AuNP solution was homogenized by an ultrasonic homogenizer (Hielscher, Germany) for 5 min adjacent to ice. The characteristics of the AuNPs were investigated by Dynamic Light Scattering (DLS), Scanning Electron Microscope (SEM), and a multi-mode reader. The solution was stored in a dark container at room temperature.

### 2.6 AuNP- antibody aggregation test

An AuNP solution with pH 9 was set by dropwise adding 100 mM borate buffer (pH = 9). A dilution series of the *anti*-Digoxigenin (Dig) antibody (250, 200, 150, 100, 50, and 0 μg/ml) was prepared. The test was performed in a 96-well plate with two replicates, and 150 μl of AuNP solution (pH = 9) was added to each desired well. Next, 10 μl of each anti-Dig antibody (RRID: AB_304362) dilution was added to the wells in increasing order of the antibody concentration, and the plate was shaken for 20 min at 450 rpm. Subsequently, 20 μl of 10 % NaCl solution was added to each well, and the plate was shaken for 5 min at 450 rpm [10,11]. Finally, the optical absorption of each well was read by a multi-mode reader in the range of 400–700 nm with 5 nm intervals.

### 2.7 Preparation of anti-Dig antibody-conjugated AuNP

The pH of the AuNP solution was adjusted to 9 with 100 mM borate buffer (pH = 9). 100 μl of 250 μg/ml anti-Dig antibodies were added to 1.5 ml of the AuNP solution and the antibody-AuNP mixture was incubated for 20 min at 650 rpm. Then, 100 μl of 1 mg/ml BSA was added to the solution and the shaking process was continued for an extra 20 min at 650 rpm. The mixture was centrifuged at 14,000 rpm for 20 min at 4 °C and the supernatant was removed. The pellet of AuNP-antibody was suspended in 500 μl conjugation pad buffer (a PBS solution containing 5 % sucrose (W/V), 1 % BSA (W/V), and 0.5 % Tween-20 (V/V)) [11,12]. Finally, SEM imaging was performed to investigate the binding of antibodies to the AuNPs' surface.

### 2.8 Fabrication of AuNP-based LFA

The sample pad was treated with sample pad buffer (a solution of PBS buffer containing 0.5 % BSA (W/V) and 0.05 % Tween-20 (V/V)) and dried at 37 °C. The conjugated pad was also immersed in the AuNP/anti-Dig conjugate solution and completely dried in the incubator. The nitrocellulose membrane was pasted on the backing card, and control and test points were loaded on it with 1 mg/ml anti-anti-Dig antibody (RRID: AB_956005/diluted in phosphate buffer 0.01 M and pH = 7.4) and 10 mg/ml Streptavidin (diluted in phosphate buffer 0.01 M and pH = 7.4), respectively. Then, it was dried in the incubator at 37 °C [11]. Absorbent and conjugate pads were placed on the backing card overlapping with the nitrocellulose membrane and finally, the sample pad was pasted as the last part (Fig. S1). All pads were cut with a width of 5 mm (Table S2).

### 2.9 LFA test

The RT-LAMP reaction was performed using WarmStart LAMP Kit (NEB, USA) and FIP, BIP, B3, F3, LF-Dig, and LB-Biotin primers for 45 min at 65 °C. The reaction was conducted on the swab samples (in VTM) that had already been heated at 65 °C for 10 min. To perform the LFA test, 1 μl of RT-LAMP reaction products was used along with 100 μl of running buffer (PBS solution including 1 % Tween-20 (V/V) [13]), and the test result was evaluated after 15 min. Moreover, 2.5 μl of RT-LAMP products were employed for 2 % agarose gel electrophoresis. Finally, gel and strip results were compared.

### 2.10 Statistical analysis

The obtained data were processed using Excel 2019. With the results obtained during colorimetric RT-LAMP and RT-LAMP-LFA diagnostic tests, the sensitivity and specificity of these two methods were calculated with the help of the following two formulas. To validate the outcome and obtain the 95 % confidence interval (95 % C.I.), the calculations were performed on MedCalc (RRID: SCR_015044) as well (https://www.medcalc.org/calc/diagnostic_test.php). The Image J software (RRID: SCR_003070) was employed for precise analysis of the RT-LAMP-LFA results.

$$\text{Sensitivity}\;\left(\%\right)=\frac{\text{Number}\;\text{of}\;\text{True}\;\text{Positives}}{\text{Number}\;\text{of}\;\text{True}\;\text{Positives}+\text{Number}\;\text{of}\;\text{False}\;\text{Negatives}}\times 100$$

$$\text{Specificity}\;\left(\%\right)=\frac{\text{Number}\;\text{of}\;\text{True}\;\text{Negatives}}{\text{Number}\;\text{of}\;\text{True}\;\text{Negatives}+\text{Number}\;\text{of}\;\text{False}\;\text{Positives}}\times 100$$

### 2.11 Ethics approval and consent to participate

All the procedures were performed in accordance with the ethical standards of the Ethics Committee of the University of Isfahan, Isfahan, Iran (Approval ID: IR.UI.REC.1399.074), and with the Declaration of Helsinki and its later amendments or comparable ethical standards. There were no consent forms as the collected swab specimens were leftovers from routine viral diagnostic testing, and no identity information was provided for the samples.

## 3 Results

### 3.1 Colorimetric RT-LAMP versus RT-qPCR

In the current study, the results of the RT-qPCR method were expressed as cycle threshold (Ct) values and classified as positive (Ct ≤ 37), and negative (Ct > 37) based on the PowerChek™ 2019-nCoV Real-time PCR Kit instruction. Also, the shift from pink to yellow/orange indicates a positive result in the colorimetric RT-LAMP assay targeting ORF8 gene.

### 3.2 Isolated RNA-based SARS-CoV-2 diagnosis

**Fig. 1:**
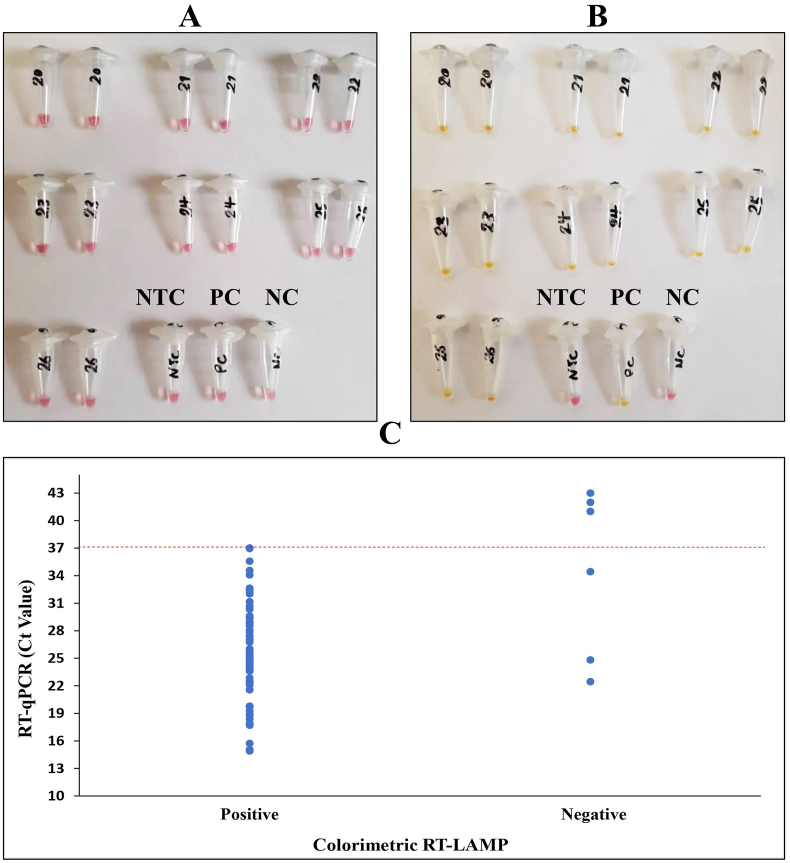
Colorimetric RT-LAMP reaction results for extracted RNAs. (A) Samples before the reaction. (B) Samples after the reaction. The shift from pink to yellow/orange indicates a positive result. The numbers on the microtubes indicate the number of samples. NTC: Non-Template Control, PC: Positive Control, and NC: Negative Control. (C) The dashed line indicates the border between positive and negative results in the RT-qPCR method. According to the chart, the true and false positive and negative results in the colorimetric RT-LAMP method can be observed. (For interpretation of the references to color in this figure legend, the reader is referred to the Web version of this article.)

**Fig. 2:**
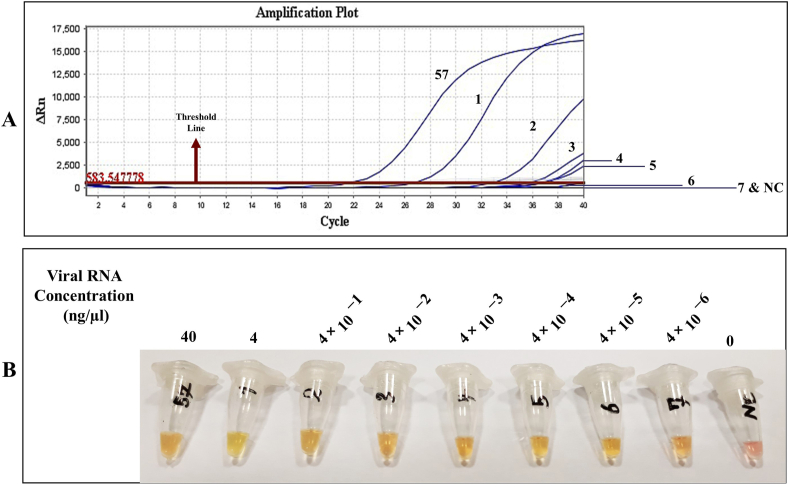
Investigation of the limit of detection (LoD) in the RT-qPCR and colorimetric RT-LAMP methods. A dilution series was prepared from a positive clinical sample. (A) All samples except 6, 7, and Negative Control (NC) had Ct ≤ 37. (B) All of them turned yellow/orange color (Positive) except the NC. (For interpretation of the references to color in this figure legend, the reader is referred to the Web version of this article.)

To evaluate the diagnostic accuracy of the colorimetric RT-LAMP technique for COVID-19 compared to the routine RT-qPCR method, we first tested two described assays on viral isolated RNA from swabs. The colorimetric RT-LAMP assay was performed using specific primers targeting the ORF8 gene. Finally, out of 60 positive samples, 57 identified true positives and three false negatives. Also, three out of the four negative samples were true negatives, and the remaining was false positives (Fig. 1 and Table 1). This isothermal-based colorimetric method had a sensitivity of 95 % (95 % C.I: 86.08 %–98.96 %) and specificity of 75 % (95 % C.I: 19.41 %–99.37 %). To determine the LoD of the two described methods, we prepared seven 10-fold serial dilutions of the original sample ranging from 40 to 4 $\times 10^{-6}$ ng/μl. According to Fig. 2, it can be concluded that the colorimetric RT-LAMP can detect until the concentration of 4 $\times 10^{-6}$ ng/μL of viral RNA while the RT-qPCR can only diagnose the viral RNA until the concentration of 4 $\times 10^{-4}$ ng/μL. Therefore, the LoD for the colorimetric RT-LAMP was 100-fold superior to RT-qPCR.

**Table 1:** Comparison of RT-qPCR cycle threshold (Ct) values to colorimetric RT-LAMP results.

Result	Ct Value RT-qPCR	Colorimetric RT-LAMP
**Positive**	<23	19 out of 20 (95 %)
	23–30	26 out of 28 (92.85 %)
	30–37	12 out of 12 (100 %)
**Total Positives**	≤ 37	57 out of 60 (95 %)
**Negative**	>37	3 out of 4 (75 %)

### 3.3 The viral detection relying on direct RT-LAMP

**Fig. 3:**
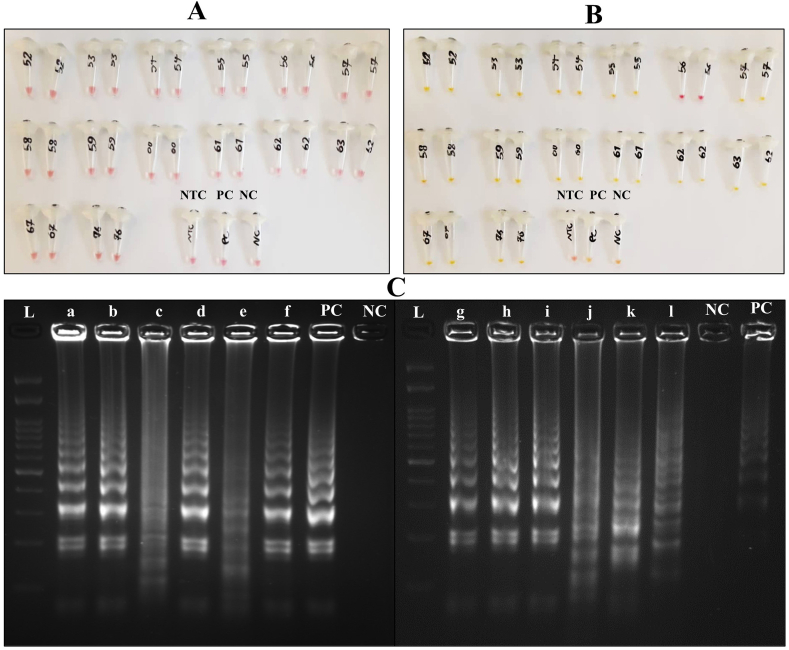
Colorimetric RT-LAMP reaction results for some non-extracted RNAs. (A) Samples before the reaction. (B) Samples after the reaction. The numbers on the microtubes indicate the number of samples. The shift from pink to yellow/orange indicates a positive result. (C) Confirmation of ORF8 gene amplification RT-LAMP products using 2 % agarose gel electrophoresis. The samples were randomly selected from extracted RNA (a, b, c, g, h, and i) and non-extracted samples (d, e, f, j, k, and l) together with the positive and negative controls of the reactions. NTC: Non-Template Control, PC: Positive Control, NC: Negative Control, and L: DNA Ladder. (For interpretation of the references to color in this figure legend, the reader is referred to the Web version of this article.)

Due to the importance of time in COVID-19 detection, minimal sample processing is required. Hence, we focused on a swab-to-RT-LAMP assay directly without the requirement for an RNA extraction step, and the diagnostic performance of this assay was investigated in this approach on 14 swab samples that tested positive by RT-qPCR (Ct as reference). As shown in Fig. 3B, except for one, the rest of the specimens revealed the color shift very well.

### 3.4 The validation of the RT-LAMP products

To ensure the colorimetric RT-LAMP amplification process, several RT-LAMP products were randomly selected among extracted RNA samples and original ones, and along with positive and negative controls of RT-LAMP reaction, were loaded on 2 % agarose gel for observing the laddered banding patterns. According to Fig. 3C, all the samples, including positive controls, showed the target gene amplification well on the gel.

### 3.5 AuNP-based RT-LAMP-LFA

To use the RT-LAMP method for faster detection of SARS-CoV-2 and develop a so-called point-of-care (POC) tool, this technique was combined with AuNP-based LFA so that the amplified products of the ORF8 gene could be more accurately detected, with higher sensitivity and specificity, compared to the colorimetric RT-LAMP.

### 3.6 The conjugation of AuNP with anti-Dig

**Fig. 4:**
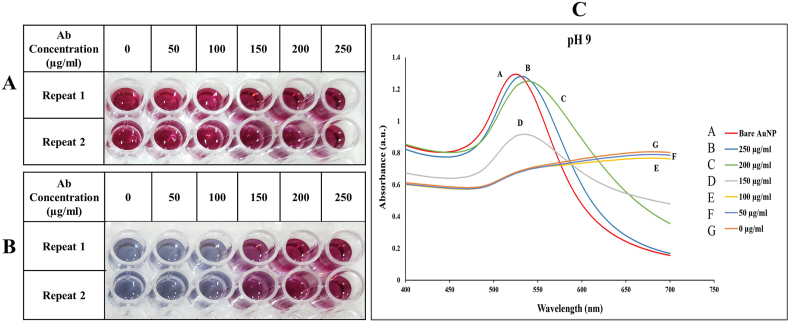
The results of the Aggregation test. (A) Before and (B) after the aggregation test. The wells corresponding to the concentration of 250 μg/ml are left with minimal color change that is because of complete conjugation in this concentration. (C) Absorption graphs of gold nanoparticles (AuNPs) with different concentrations of anti-Dig antibody. The concentration graph of 250 μg/ml shows the lowest displacement compared to the bare AuNPs graph. (For interpretation of the references to color in this figure legend, the reader is referred to the Web version of this article.)

**Fig. 5:**
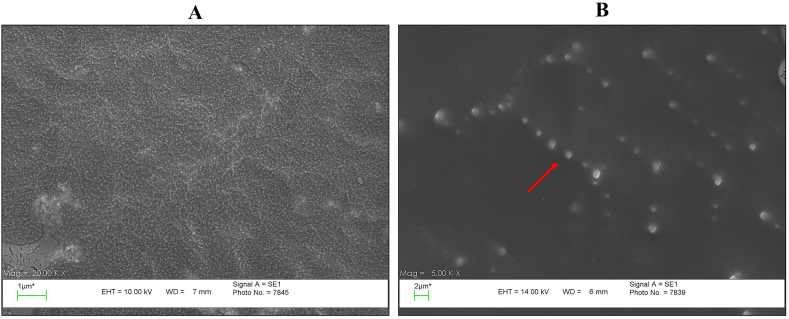
Photographs of gold nanoparticles (AuNPs) using the SEM microscope. These pictures depict two different steps of AuNPs: (A) Before conjugation with anti-Dig antibodies with an average diameter below 50 nm. (B) After conjugation with the antibody. The increase in the hydrodynamic diameter of AuNPs after conjugation with antibodies can be seen. The red arrow shows one conjugated AuNP. (For interpretation of the references to color in this figure legend, the reader is referred to the Web version of this article.)

The synthesis of AuNPs with suitable diameter is a critical step in developing an AuNP-based LFA, and the widely used range of AuNP in LFA is 20–40 nm [14]. According to DLS results (Table S3), the average diameter of synthesized AuNPs was 42.9 nm, and more investigations by SEM imaging and absorption spectrum confirmed this result (Fig. 5A and Fig. S2). The Turkevich method is a commonly used method to synthesize spherical AuNPs. The wine-red color and a single absorption band in the visible spectrum are the distinctive features of spherical AuNPs, which can be observed in Fig. 4 and Fig. S2B [15]. To obtain the appropriate concentration of anti-Dig antibody which prevented salt-induced AuNP accumulation, an aggregation test was performed. Based on the objective observations and absorption spectrum analysis, the concentration of 250 μg/ml was selected as the most suitable concentration for conjugating to AuNPs (Fig. 4). Furthermore, to confirm the binding of antibodies to AuNPs, SEM imaging was applied on two AuNP samples before and after conjugation with anti-Dig antibodies (Fig. 5).

### 3.7 Performance evaluation of the RT-LAMP-LFA method

**Fig. 6:**
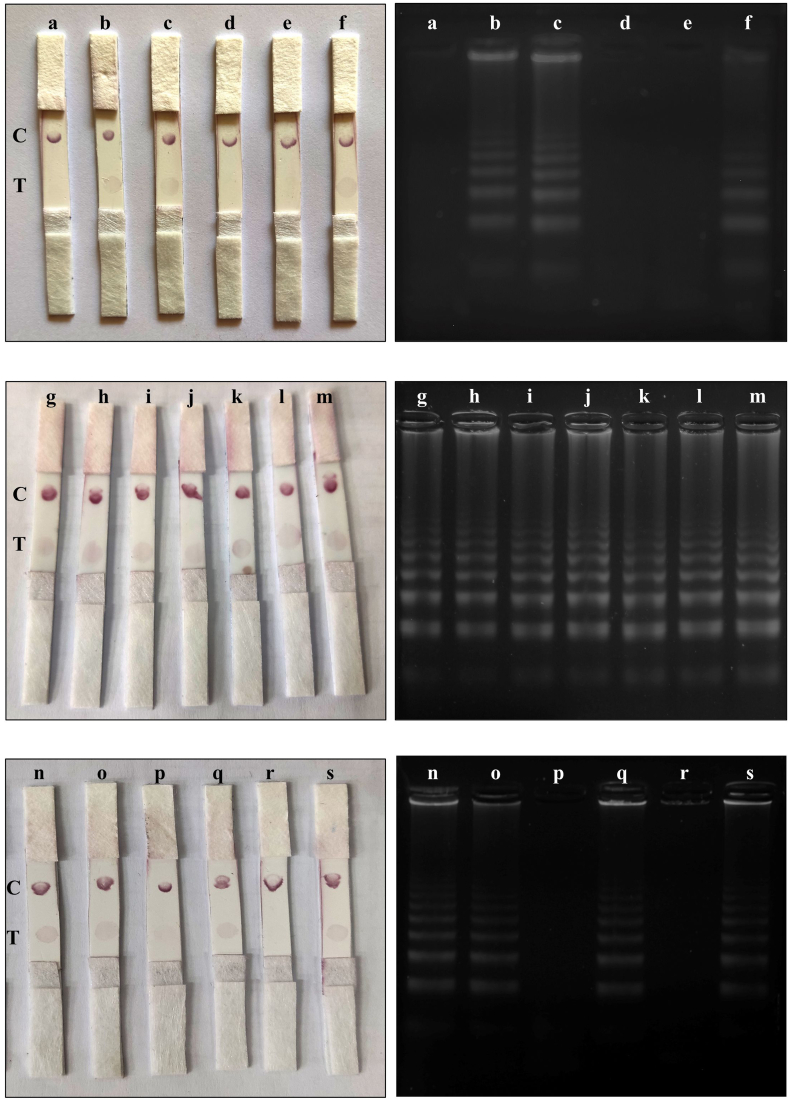
The results of gel electrophoresis and LFA. The ladder-shaped pattern on the gel represents the products resulting from the amplification of the target gene during the RT-LAMP reaction. The signals in the control and test areas on the test strips indicate the positive sample and the signal only in the control section indicates the negative sample. All samples are positive apart from (a), (d), (e), (p), and (r), although the (p) showed a false positive on the LFA. C: control point and T: test point.

To visualize the RT-LAMP products and boost the diagnostic efficiency of this technique compared to the colorimetric technique, the direct RT-LAMP was combined with the fast and accurate LFA method. After performing the direct RT-LAMP reaction on the oro-nasopharyngeal swab samples, the amplified products were loaded on 2 % agarose gel to investigate the proliferation process and then analyzed on the strip tests. After that, the gel and test strip results were compared together (Fig. 6). A total of 66 samples were examined. The LFA results were determined as red signals in the control and test sections for positive samples and red signals only in the control section for negative samples. Out of 44 positive and 22 negative samples confirmed by gel electrophoresis, 44 positive and 17 negative samples were also confirmed by LFA. Five false positives were recorded for LFA. According to that, our RT-LAMP-LFA kit has 100 % sensitivity (95 % C.I: 91.96 %–100.00 %) and 77.27 % specificity (95 % C.I: 54.63 %–92.18 %) for SARS-CoV-2 diagnosis. The comparison of the gel electrophoresis and LFA results is shown in Table 2.

**Table 2:** Evaluation of the sensitivity and specificity of the RT-LAMP-LFA method.

Gel	LFA		Metrics % (95 % C.I.)		
	Positive	Negative	Total	Sensitivity (%)	Specificity (%)
**Positive**	44	0	44	100 (91.96–100)	77.27 (54.63–92.18)
**Negative**	5	17	22		
**Total**	49	17	66		

## 4 Discussion

In the present study, RT-qPCR and colorimetric RT-LAMP techniques were compared together in the first step to investigate the RT-LAMP technique as an alternative method for the detection of SARS-CoV-2. By comparing the results of colorimetric RT-LAMP and RT-qPCR (based on isolated RNA), the specificity and sensitivity of 75 % (95 % C.I: 19.41 %–99.37 %) and 95 % (95 % C.I: 86.08 %–98.96 %) were obtained, respectively. It should be noted that the low percentage of specificity was fundamentally associated with the small number of negative samples analyzed in our study. The LoD of the colorimetric RT-LAMP and RT-qPCR methods was compared together using a dilution series of viral RNA extracted from a positive clinical sample. According to this comparison, the LoD of the RT-LAMP technique was calculated 100 times higher than the RT-qPCR technique. As mentioned, this study used only one primer set of the ORF8 gene. Therefore, it has enjoyed higher accuracy compared to the multiplex RT-LAMP method [16].

Moreover, the colorimetric RT-LAMP technique was directly applied to some crude samples to investigate the direct diagnostic ability of this method. The findings showed that it is possible to eliminate the time-consuming and laborious step of RNA extraction. In this study, no chemical treatment was used to lyse cells and inactivate the virus, unlike other studies that utilized substances like NaOH [[17], [18], [19]]. In our research, the samples were just heated for 10 min at 65 °C before the RT-LAMP reaction. Consequently, the reaction time and cost were significantly reduced. Finally, it was found that even with the contamination from the lysed samples in the reaction tube, RT-LAMP is still capable of amplifying the target gene with high sensitivity (13 out of 14 positive samples confirmed by RT-qPCR with direct colorimetric RT-LAMP were also diagnosed as positive). The only false negative result in the process of colorimetric RT-LAMP using pre-heated samples might be due to an incomplete lysis procedure at 65 °C. Due to the high viscosity of the swab sample, it is estimated that a longer incubation time may have been necessary to ensure complete lysis of the cells and viral particles. Also, as colorimetric RT-LAMP can be sensitive to pH fluctuations, we opted for LFA as a visualizing method to observe direct RT-LAMP results. However, when we calculated the sensitivity only for the 14 samples tested by direct colorimetric RT-LAMP using the formula mentioned in the “Statistical Analysis” section, it was found to be 92.85 %, which is acceptable. Additionally, our direct RT-LAMP-LFA was able to correctly diagnose all true positives with a 100 % sensitivity rate (44 out of 44).

ORF8 is considered a key factor in the virus's pathogenicity and an important therapeutic target [20]. The ORF8 gene encodes an accessory protein in SARS-CoV-2, which leads to interference in the function of immune responses in the host cell. Compared to other proteins of this virus, this protein has the least homology with other coronaviruses such as SARS-CoV [21,22]. The findings indicated that ORF8 has diverse functions primarily aimed at countering the immune responses of the host and impacting various biological processes [20]. However, in comparison with other virus genes such as N [23,24], ORF1ab [[25], [26], [27]], S [28,29], and RdRp [1,30], this gene has received less attention in terms of diagnosis. Moreover, based on the Mautner et al. study, the ORF8 gene demonstrated better sensitivity in diagnosing positive swab samples via direct RT-LAMP when Ct was lower than 35, compared to the N gene [9]. Like other genes such as S, N, and M, ORF8 can also undergo mutations during SARS-CoV-2 evolution. Nonetheless, the frequency of ORF8 mutations is comparatively lower worldwide, particularly in Asia [31]; however, the so-far-known mutations in the ORF8 gene have no significant effect on the selected LAMP primer set. The most frequent mutation is L84S, which is not in the critical sequence for LAMP primers attachment (Table S5 [32] and Fig. S1 [9]). According to Mautner et al. results in using ORF8 LAMP primers for SARS-CoV-2 diagnosis, no cross-reactivity was observed in the detection of SARS-CoV-2 among 20 other respiratory pathogens, including SARS-CoV, Coronaviruses 229E, HKU-1, NL63, OC43, and Parainfluenza virus type 1–4 (Table 2 [9]). This finding implicates the high specificity of the selected primer set for ORF8. The primary studies to detect SARS-CoV-2 targeting the ORF8 region were carried out using RT-LAMP on a small number of clinical specimens, and synthetic samples were investigated in these studies [9,33]. However, we performed the colorimetric RT-LAMP method on 64 oro-nasopharyngeal swab samples (60 positive samples and 4 negative samples) by targeting the ORF8 gene.

Today, visualizing the results of diagnostic reactions is in turn, essential and efficient. The LFA is a serology assay-based method that is extensively employed for revealing diagnosis results. Therefore, the development of a fast LFA method based on direct RT-LAMP (RT-LAMP-LFA) was contemplated as the focal point of this study. This diagnostic technique is considered an affordable approach compared to the RT-qPCR test and can perform the diagnosis earlier and with more accuracy. In this step, 66 oro-nasopharyngeal swab samples (44 positive samples and 22 negative samples) immersed in VTM were used. In a study conducted by Jang et al. their developed RT-LAMP-LFA method was examined on RNA samples extracted from 23 nasopharyngeal swab samples [34]. Unlike this study, we prepared all strip test components separately and then assembled them, and only 1 μl of direct RT-LAMP products was used for the LFA. On the other hand, to get closer to the “point of care” objective, the RNA extraction process was completely omitted, contrary to other research [4,35,36]. Agarwal et al. utilized crude throat swabs, routinely heated at 56 °C for 30 min before RNA extraction for RT-qPCR and a thermocycler to conduct the RT-LAMP reaction. However, we only heated the samples at 65 °C for 10 min and used a simple thermoblock, making the test faster, more affordable, and easier to perform [37]. Alhamid et al. developed a new RT-LAMP technique using five primers to optimize specificity & reduce false positives for the detection of the E gene on RNA samples, which makes the differences noticeable compared to our work using ORF8 and crude samples [38]. Two other studies targeted different genes rather than ORF8 through RT-LAMP. These studies introduced isolated RNAs to the RT-LAMP reaction. One of these studies showed low sensitivity for samples with Ct higher than 30 (81.3 % and 76.5 % for Ct > 30 and Ct > 35, respectively) [39]. Nonetheless, our sensitivity for colorimetric RT-LAMP and direct RT-LAMP-LFA was 95 % and 100 %, respectively, for positive samples with Ct lower than 37. The other study utilized a thermocycler for RT-LAMP amplification and a spectrophotometer for absorbance reading. The entire process took 5–6 h from sample arrival to results announcement [40]. In contrast, we used a simple thermoblock for both sample lysis and RT-LAMP amplification to keep the process simple and cost-effective. Moreover, the whole process from sample collection to direct RT-LAMP-LFA test interpretation takes only 75–90 min. In contrast to our study, Hoffmann et al. presented a direct RT-LAMP method that uses the RP gene to detect SARS-CoV-2. This approach simplifies and speeds up the diagnosis process by using crude samples. However, they only tested the method on positive samples with Ct values lower than 30 [41], whereas we demonstrated acceptable sensitivity and specificity for positive samples with Ct values lower than 37. There have been no published studies yet on a technique for diagnosing ORF8 by combining RT-LAMP with LFA without RNA extraction. According to the obtained results, our direct RT-LAMP-LFA method showed 77.27 % (95 % C.I: 54.63 %–92.18 %) specificity and 100 % (95 % C.I: 91.96 %–100.00 %) sensitivity for ORF8 gene detection in oro-nasopharyngeal swab samples. Obviously, by solving the limitations of this study and doing further research, a higher specificity can be achieved; the limitations such as the small number of tested negative samples, the lack of testing on other types of viral respiratory pathogens, and no investigation of the effect of VTM on the performance of the RT-LAMP-LFA reaction.

## 5 Conclusion

This study presents an RT-LAMP-LFA tool for rapid and high-sensitivity detection of SARS-CoV-2 based on the viral ORF8 gene. In this method, the identification process is performed directly on heat-treated clinical samples to eliminate the time-consuming RNA extraction, which subsequently reduces the entire time required from sampling to test interpretation. In such a way that the whole procedure only demands 75–90 min to complete, compared to a 4-h RT-qPCR detection procedure. Assuredly with further optimization and research, this tool, as a point-of-care method, can conveniently substitute the RT-qPCR technique.

## CRediT authorship contribution statement

**Negar Sadeghi:** Writing – original draft, Visualization, Validation, Investigation, Formal analysis. **Neda Shirazi:** Writing – original draft, Visualization, Validation, Investigation, Formal analysis. **Moein Dehbashi:** Writing – review & editing, Validation, Methodology, Investigation, Conceptualization. **Bahareh Maleki:** Writing – original draft. **William C. Cho:** Writing – review & editing, Validation. **Zohreh Hojati:** Writing – review & editing, Validation, Supervision, Resources, Project administration, Methodology, Funding acquisition, Conceptualization.

## Availability of data and materials

All data generated or analyzed during this study are included in this published article and its supplementary information file.

## Ethics approval and consent to participate

All the procedures were performed in accordance with the ethical standards of the Ethics Committee of the University of Isfahan, Isfahan, Iran (Approval ID: IR.UI.REC.1399.074), and with the Declaration of Helsinki and its later amendments or comparable ethical standards. There were no consent forms as the collected swab specimens were leftovers from routine viral diagnostic testing, and no identity information was provided for the samples.

## Funding

This work was supported by the 10.13039/501100007087University of Isfahan (Project NO.: 1443/J/S99) and the National Institute of Genetic Engineering and Biotechnology (Project NO.: 99/1443).

## Declaration of competing interest

The authors declare the following financial interests/personal relationships which may be considered as potential competing interests.

Zohreh Hojati reports financial support was provided by National Institute of Genetic Engineering and Biotechnology. If there are other authors, they declare that they have no known competing financial interests or personal relationships that could have appeared to influence the work reported in this paper.

## Data Availability

All data generated or analyzed during this study are included in this published article and its supplementary information file.

## List of abbreviations

Severe acute respiratory corona virus 2 (SARS-CoV-2); Reverse transcription quantitative polymerase chain reaction (RT-qPCR); Reverse transcription loop-mediated isothermal amplification (RT-LAMP); Lateral flow assay (LFA); Open reading frame 8 (ORF8); RNA-dependent RNA polymerase (RdRp); Coronavirus disease 2019 (COVID-19); Nucleic acid amplification technique (NAAT); Isothermal nucleic acid amplification technique (iNAAT); Viral transport medium (VTM); Forward primer 3 (F3); Backward primer 3 (B3); Forward inner primer (FIP); Backward inner primer (BIP); Loop forward primer (LF); Loop backward primer (LB); Limit of detection (LoD); Gold nanoparticle (AuNP); Dynamic light scattering (DLS); Scanning electron microscope (SEM)); Bovine serum albumin (BSA); Phosphate-buffered saline (PBS); Weight per volume (W/V); Volume per volume (V/V); Digoxygenin (Dig); Immunoglobulin G (IgG); Cycle threshold (Ct); Confidence interval (C.I.); Non-template control (NTC); Positive control (PC); Negative control (NC); Point-of-care (POC).
