# Supplementary material for: Development of a rapid LFA test based on direct RT-LAMP for diagnosis of SARS-CoV-2
Source: Pract Lab Med. 2024 Oct 18;42:e00437. doi: 10.1016/j.plabm.2024.e00437 (PMC11565418; doi:10.1016/j.plabm.2024.e00437)
Supplement: Multimedia component 1 [file mmc1.docx]

**Supplementary information**


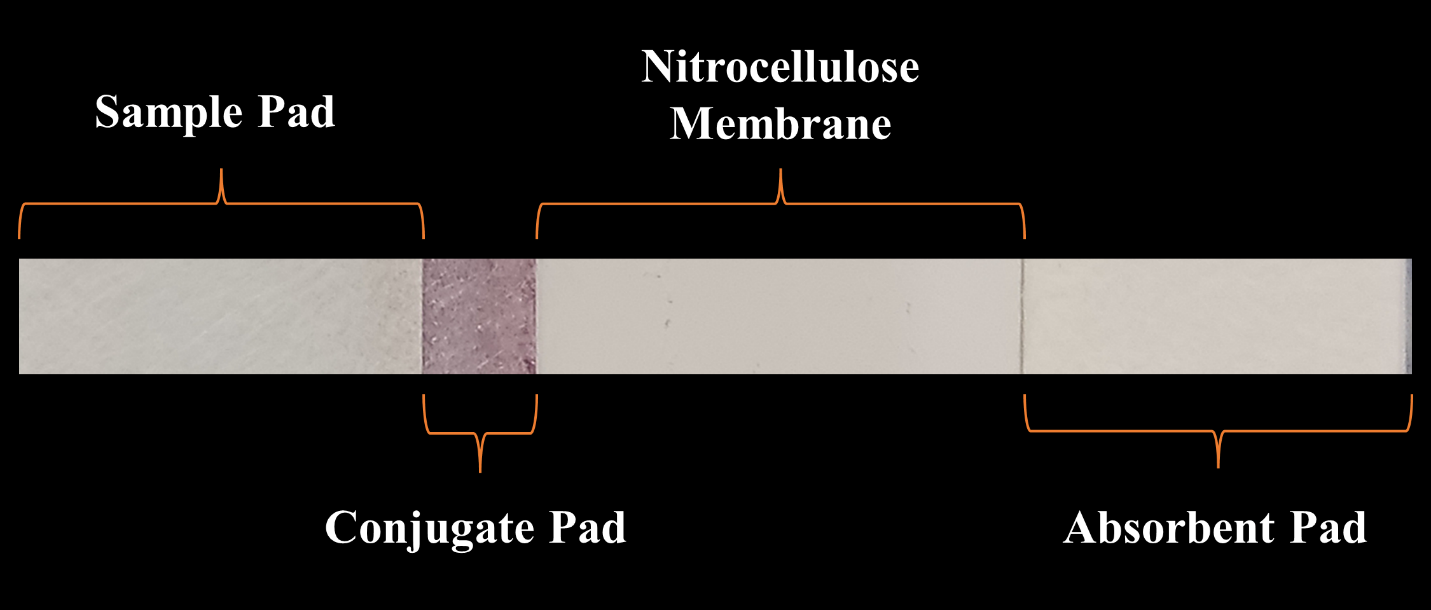


**Supplementary Figure 1. The structure of a strip test.** After treatment with buffers, loading test and control points, and drying, the pads were finally pasted on the backing card.

**
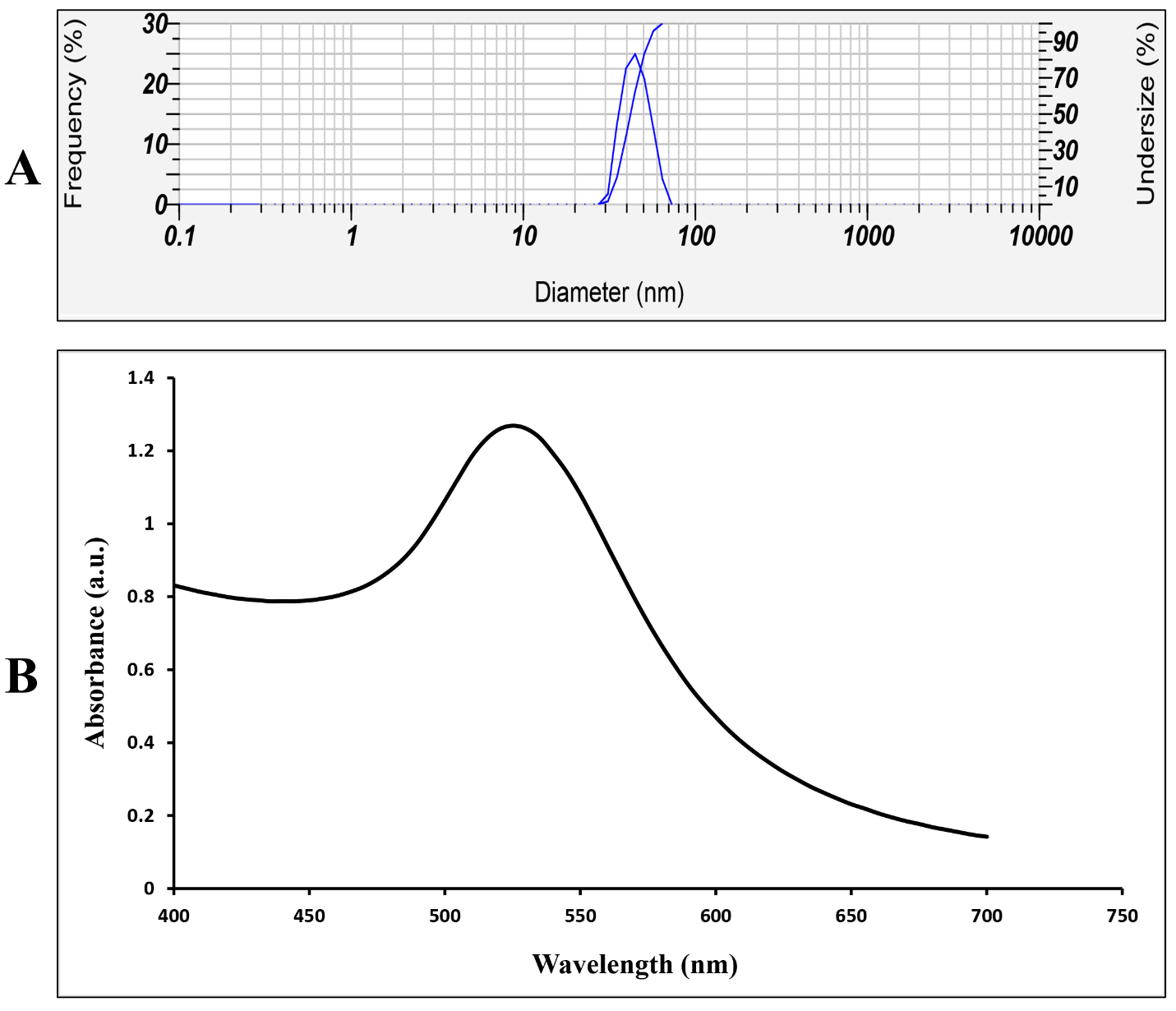
**

**Supplementary Figure 2. Investigation of the synthesized gold nanoparticle (AuNP) features (A)** Dynamic light scattering (DLS) result of AuNPs. The bell-shaped graph shows the normal distribution of the nanoparticles size and its single peak indicates the purity of the solution. (**B)** Absorption diagram of AuNPs in the range of 400-700 nm. The maximum absorption value is seen at the wavelength of 525 nm.

**Supplementary Table 1.** The sequence of used primers

| **Target Gene** | **Primer** | **Sequence 5′–3′** |
| --- | --- | --- |
| **ORF8** | ORF8-F3 | ACT TGT CAC GCC TAA ACG |
|  | ORF8-B3 | CTA CCC AAT TTA GGT TCC TGG |
|  | OFR8-FIP | AGG ACA CGG GTC ATC AAC TAC AAG CTG CAT TTC ACC AAG AA |
|  | ORF8-BIP | AGG AGC TAG AAA ATC AGC ACC TAT GGG TGA TTT AGA ACC AGC |
|  | ORF8-LF | TGG TTG ATG TTG AGT ACA TGA C |
|  | ORF8-LB | AAT TGA ATT GTG CGT GGA TGA G |
|  | ORF8-LF-Dig | Digoxigenin- TGG TTG ATG TTG AGT ACA TGA C |
|  | ORF8-LB-Biotin | Biotin- AAT TGA ATT GTG CGT GGA TGA G |

**Supplementary Table 2.** The size of the pads used in the manufacture of the LFA strip

| **Pad’s name** | **Length** (mm) | **Width** (mm) |
| --- | --- | --- |
| Backing Card | 60 | 5 |
| Nitrocellulose membrane | 25 | 5 |
| Absorbent Pad | 15 (2 overlapping with membrane) | 5 |
| Conjugate Pad | 7 (2 overlapping with membrane) | 5 |
| Sample Pad | 18 (1 overlapping with conjugate pad) | 5 |

**Supplementary Table 3.** DLS result of synthesized gold nanoparticle (AuNP).

| **Peak No.** | **S.P. Area Ratio** | **Mean** (nm) | **S.D.** (nm) | **Mode** (nm) |
| --- | --- | --- | --- | --- |
| 1 | 1.00 | 42.9 | 7.5 | 41.9 |
| 2 | - | - | - | - |
| 3 | - | - | - | - |
| Total | 1.00 | 42.9 | 7.5 | 41.9 |

Note: The closeness of the mean to the mode indicates a normal distribution of particle sizes. S.D.: Standard Deviation.
